# An observational study of children with sickle cell disease in Kilifi, Kenya

**DOI:** 10.1111/j.1365-2141.2009.07771.x

**Published:** 2009-09

**Authors:** Manish Sadarangani, Julie Makani, Albert N Komba, Tolu Ajala-Agbo, Charles R Newton, Kevin Marsh, Thomas N Williams

**Affiliations:** 1 Centre for Geographical Medicine (Coast) (CGMRC), Kenya Medical Research Institute (KEMRI)Kilifi, Kenya; 2 Department of Haematology and Blood Transfusion, School of Medicine, Muhimbili University of Health and Allied SciencesDar-es-Salaam, Tanzania; 3 School of Medicine, University of SouthamptonSouthampton; 4 Nuffield Department of Clinical Medicine, University of OxfordOxford, UK; 5 INDEPTH NetworkAccra, Ghana

**Keywords:** Africa, children, out-patient, sickle cell

## Abstract

Globally, sickle cell disease (SCD) has its highest prevalence and worst prognosis in sub-Saharan Africa. Nevertheless, relatively few studies describe the clinical characteristics of children with SCD in this region. We conducted a prospective observational study of children with SCD attending a specialist out-patient clinic in Kilifi, Kenya. A total of 124 children (median age 6·3 years) were included in the study. Splenomegaly was present in 41 (33%) subjects and hepatomegaly in 25 (20%), both being common in all age groups. A positive malaria slide was found at 6% of clinic visits. The mean haemoglobin concentration was 73 g/l, compared to 107 g/l in non-SCD controls (*P* < 0·001). Liver function tests were elevated; plasma bilirubin concentrations were 46 μmol/l and aspartate aminotransferase was 124 iu/l. Forty-eight (39%) children were admitted to hospital and two died. Children with SCD in Kilifi have a similar degree of anaemia and liver function derangement to patients living in developed countries, but splenomegaly persists into later childhood. The prevalence of malaria was lower than expected given the prevalence in the local community. This study provides valuable data regarding the clinical characteristics of children living with SCD in a rural setting in East Africa.

Recent estimates suggest that 300 000 children are born with sickle cell disease (SCD) worldwide every year and that three-quarters of these births occur in sub-Saharan Africa (Diallo & Tchernia, 2002; World Health Organization [WHO] 2006). Nevertheless, descriptions of the natural history and clinical spectrum of SCD in Africa are surprisingly scarce and, as a result, most treatment recommendations for the management are based on studies conducted in resource-rich countries.

With improved access to medical care, socio-economic development and better education, survival of patients with SCD is improving in a number of African countries (Corachan *et al*, 1979; Elamin, 1980; Knox-Macaulay, 1983; Athale & Chintu, 1994; Koko *et al*, 1998). Baseline data regarding the clinical course of SCD in this context are therefore required to inform evidence-based decisions regarding appropriate management. This paper describes the clinical, anthropometric and laboratory characteristics of a cohort of children with SCD attending a specialist out-patient clinic in a rural part of Kenya.

## Methods

### Study location

The study was conducted at Kilifi District Hospital (KDH), which is located 60 km to the north of Mombasa on the Kenyan coast. KDH is the main district level government inpatient facility, serving a community of about 100 000 children under 15 years of age. Kilifi is one of the poorest districts in Kenya, the economy being dominated by subsistence agriculture (Snow *et al*, 1993). The prevalence of sickle cell trait (HbAS) in this area is 15% (Williams *et al*, 2005) and homozygosity for HbS (HbSS) is the only form of SCD that has been documented in the local community.

### Study subjects

Our study involved children attending the SCD outpatient clinic at KDH. All subjects were <14 years old at the time of recruitment and had the diagnosis of homozygous SCD (HbSS) confirmed by alkaline haemoglobin electrophoresis. Data on these children were collected prospectively over a 19-month period between 1 June 2003 and 31 December 2004. Non-SCD controls were members of a longitudinal cohort study designed to study the immunology of malaria as described previously (Urban *et al*, 2006). Anthropometric data from control subjects were collected in October 2003 (Nyakeriga *et al*, 2004) and blood counts performed during a cross-sectional survey in October 2004.

### Routine follow up

Children were reviewed routinely in the outpatient clinic on a 3-monthly basis and were encouraged to attend between appointments in the event of intercurrent illness. Children who failed to attend the clinic for more than 6 months were traced in the community, and considered lost to follow-up if they could not be located. Routine management included patient education, folic acid supplementation and anti-malarial chemoprophylaxis with proguanil. Malnutrition and anaemia were managed according to WHO guidelines (WHO 2005). Parents and carers of malnourished children were advised regarding feeding and those with severe malnutrition were admitted for in-patient hospital management. Children with iron deficiency were offered ferrous sulphate and mebendazole. Penicillin is not recommended routinely for prophylaxis against invasive bacterial diseases in patients with SCD in Kenya, and pneumococcal vaccines were not available at the time of the study. Anthropometric indices (weight, height/length and mid-upper arm circumference) were measured at every clinic visit using standard methods (Nyakeriga *et al*, 2004). Routine clinical assessments (including the measurement of trans-cutaneous oxygen saturation) were recorded on standard proformas. Routine investigations included full blood and reticulocyte counts, blood film examination for malaria parasites, renal and liver function tests and urine testing for protein, blood, glucose and nitrites. Further investigations were performed as clinically indicated. Diagnosis of suspected bacterial infection, treatment and decision to admit were based on WHO guidelines (WHO 2005). All patients were offered voluntary counselling and testing for human immunodeficiency virus (HIV). Data regarding hospital admissions were recorded on standard proformas.

### Laboratory data

Haemoglobin was analysed by electrophoresis on cellulose acetate gels using standard methods (Wild & Bain, 2006). Full blood counts were performed using a Coulter MDII-18 counter (Beckman-Coulter, Fullerton CA, USA). Thick and thin blood smears were examined for malaria parasites at ×1000 magnification using standard methods (English *et al*, 1996), and parasite densities were computed by comparison to white bloods cells. Plasma concentrations of sodium and potassium were determined using ion selective electrodes (Ciba-Corning Diagnostics, Halstead, UK) and creatinine concentrations with a Creatinine analyser (Beckman-Coulter). Liver function tests were carried out with a Selectra-E auto-analyser (Vital Scientific, AC Dieren, The Netherlands). Antibodies to HIV were tested by enzyme-linked immunosorbent assay (Vironostika, Biomerieux, France) and dipstick (Determine; Abbott Laboratories, Maidenhead, UK). Positive samples from children under 18 months of age and samples with discordant results were assayed for proviral DNA by polymerase chain reaction.

### Ethical considerations

This study was approved by the Kenyan National Research Ethics Committee. Written informed consent was obtained from the parents or guardians of all subjects in their own language.

### Data handling

All data were subjected to range and consistency checks before double-entry onto a computerised database written in FoxPro (version 6; Microsoft, Redmond, WA, USA). *Z*-scores were calculated using EpiInfo (version 3.3.2; CDC, Atlanta, GA, USA). Statistical analyses were conducted using the Statistical Package for the Social Sciences (spss) (version 11.5; SPSS, Chicago, IL, USA). Means of normally distributed data were compared using the Student’s *t*-test. Categorical variables were compared using Pearson’s chi-square or Fisher’s exact tests as indicated. *P* values <0·05 were considered significant.

## Results

**Fig 1:**
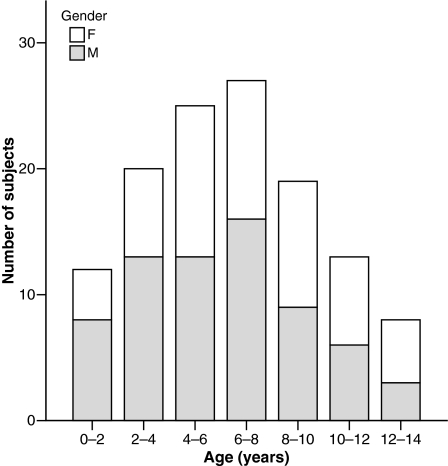
Age and gender of patients attending the SCD clinic.

A total of 124 patients were followed for a median of 13·8 months (range 0–18). Five hundred and eighty-three clinic visits were recorded during the study period (median 5, range 1–8 per patient) during a period equivalent to 118 patient-years of follow-up. Patient ages ranged from 0·8 to 13·7 years (median 6·3 years) at the time of recruitment. Sixty-eight patients (55%) were male. The age and gender distributions of the study population are shown in Fig 1. A total of 88 admissions were recorded in 48 subjects, a rate of 0·45 admissions per patient per year. Amongst patients who were admitted the median number of admissions was one (range 1–9). At 31 December 2004, 113 (91%) patients were alive, nine (7%) had been lost to follow up and two (2%) had died. One child died in hospital with an aplastic crisis and the other in the community without a cause being identified.

### Clinical findings

**Fig 2:**
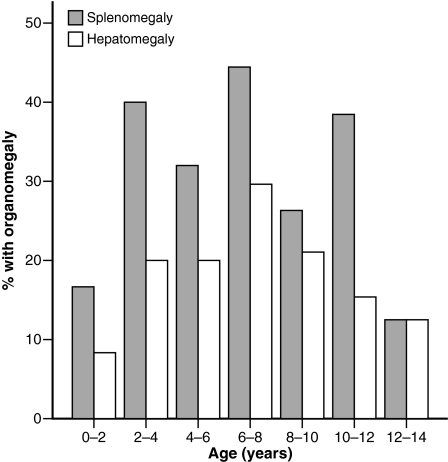
Proportion of children with organomegaly according to age.

Patients reported symptoms of illness on 130 of 583 (22%) clinic visits (Table I). There was no difference in overall frequency of symptoms at different ages (χ^2^ = 9·909, *P* = 0·194). Forty-one of 124 patients (33%) had clinically detectable splenomegaly and 25 (20%) had hepatomegaly. While both were found in all age groups, the peak prevalence for both occurred in the 6–8 year age group where 44% of patients had splenomegaly and 30% had hepatomegaly (Fig 2). Among children with a palpable spleen, the median splenic size was 3 cm below the costal margin (range 1–10 cm). In those with a palpable liver the median liver size was 2 cm (range 1–5 cm). The largest mean spleen size of 4·8 cm was seen in the 8- to 10-year age-group, and the largest mean liver size of 4·0 cm was seen in the 0- to 2-year age-group. However, overall there were no significant relationships between spleen or liver sizes and age (*P* = 0·065 and 0·672 respectively). In addition there was no relationship between splenic size and the number of episodes of malaria (*P* = 0·072), malaria parasitaemia (*P* = 0·704) or use of proguanil (χ^2^ = 3·083, *P* = 0·798). One hundred and fourteen of 124 (92%) patients reported being compliant with folic acid and proguanil prophylaxis. Bone or joint abnormalities, including swelling and tenderness, were found on 23 of 583 occasions (4%). A cardiac murmur was heard on 11 occasions (2%) (never in the same patient more than once) while skin infections were found on two occasions.

**Table I:** Symptoms reported at time of clinic visit.

	Number (% of age group) with symptoms at the time of clinic visit				
Age (years)	Any symptom	Fever	Pain	Cough or difficulty breathing	Other
0–2	8 (28)	4 (14)	2 (7)	3 (10)	5 (17)
2–4	15 (24)	4 (6)	2 (3)	5 (8)	8 (13)
4–6	28 (27)	10 (10)	5 (5)	8 (8)	12 (12)
6–8	26 (21)	8 (6)	4 (3)	4 (3)	16 (13)
8–10	32 (27)	9 (8)	8 (7)	2 (2)	19 (16)
10–12	15 (19)	2 (3)	5 (6)	1 (1)	9 (11)
12–14	4 (8)	1 (2)	1 (2)	0 (0)	2 (4)
14–16	2 (15)	0 (0)	1 (8)	0 (0)	1 (8)
Overall	130 (22)	38 (7)	28 (5)	23 (4)	72 (12)

**Fig 3:**
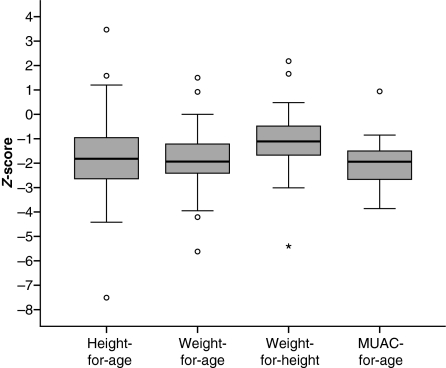
Nutritional *Z*-scores of children attending the SCD clinic. Median *Z*-scores for height-for-age, weight-for-age, weight-for-height and mid upper-arm circumference (MUAC)-for-age were −1·90 (*n* = 123), −2·00 (*n* = 124), −1·50 (*n* = 108) and −1·85 (*n* = 43) respectively. ^o^Outliers; *extremes.

The distributions of *Z*-scores for height-for-age, weight-for-age, weight-for-height and mid upper-arm circumference-for-age are illustrated in Fig 3. The mean weight-for-age and weight-for-height *Z*-scores were both significantly lower than the equivalents in the local non-SCD population (−1·86 vs. −1·47 and −1·10 vs. −0·55 respectively; *P* < 0·001). There was no significant difference in height-for-age *Z*-score between children with SCD and controls (*P* = 0·833). Fever (defined as temperature ≥37·5°C) was recorded on 68 of 583 (12%) clinic visits. Hypoxaemia (transcutaneous oxygen saturation <93%) was recorded 36 times (6%). Five patients were hypoxaemic on more than one clinic visit.

### Investigations

The haematological details of patients and controls are summarised in Table II. In patients with SCD, neither haemoglobin concentration (Hb) nor mean cell volume (MCV) varied significantly with age (*P* = 0·331 and 0·595 respectively). Hb concentrations were significantly lower (73 g/l) and MCV significantly higher (83·8 fl) in children with SCD than controls (107 g/l and 74·7 fl respectively; *P* < 0·001). The white blood cell count was also significantly raised in those with SCD compared to controls (19·2 × 10^9^/l vs. 9·3 × 10^9^/l; *P* < 0·001). A positive malaria slide was found on 37/583 (6%) occasions: 18 patients were positive on one occasion, five on two occasions and three on three occasions. The median parasitaemia in patients with a positive malaria slide was 2903/μl. Ten of 37 (27%) patients with a positive malaria slide were febrile compared with only 55 out of 535 patients (10%) with a negative malaria slide (Fisher’s exact test, *P* = 0·005).

**Table II:** Haematological parameters in SCD and non-SCD children.

	SCD		non-SCD			
Variable	Mean (SD)	*N*	Mean (SD)	*N*	Difference* (95% CI)	*P* value
Hb (g/l)	73 (13)	124	107 (14)	262	−34 (−37, −31)	<0·001
Hct (%)	23·1 (4·2)	124	32·5 (3·9)	262	−9·4 (−10·2, −8·6)	<0·001
MCV (fl)	83·8 (10·9)	124	74·7 (8·7)	262	9·1 (6·9, 111·3)	<0·001
MCHC (g/l)	318 (12)	124	329 (19)	262	−11 (−14, −8)	<0·001
WBC (×10^9^/l)	19·2 (11·6)	124	9·3 (3·5)	262	9·9 (7·8, 12·0)	<0·001
Platelets (10^9^/l)	386 (169)	124	372 (156)	262	14 (−49, 21)	0·4354
Reticulocytes (%)	11·5 (6·7)	119	N/A	–	–	–

Hb, haemoglobin; Hct, haematocrit; MCV, mean cell volume; MCHC, mean cell haemoglobin concentration; WBC, white blood cells.

* Difference between means; a positive value implies value in SCD is greater, a negative value implies value in SCD is lower.

Liver function tests were generally elevated in the study population. Mean (standard deviation, SD) total bilirubin was 46 (59) μmol/l and aspartate aminotransferase (AST) was 124 (36) iu/l. There was no significant relationship between bilirubin and age (*P* = 0·158) but AST rose with age at the rate of 2·7 iu/year (*P* = 0·005). Renal function tests (sodium, potassium and creatinine) were within normal ranges for all patients. Urine tests were abnormal on 15 of 583 occasions (3%). Proteinuria was detected in 11 children and microscopic haematuria in one. One patient tested positive for proteinuria on two separate occasions. Of the 76 patients tested for HIV only one was positive.

## Discussion

Previous descriptions of SCD from Africa have largely focused on patients in crisis. Few studies have described the clinical characteristics of patients with SCD at steady-state. Here we present data on children with SCD in attendance for routine review at a specialised out-patient clinic at Kilifi District Hospital on the Kenyan coast. While patients were also encouraged to use the clinic during illness episodes the vast majority of visits were for routine appointments and the current study, therefore, represents the clinical spectrum of SCD at steady-state in this community.

In developed countries, the spleen is often palpable in early life due to extramedullary haematopoiesis, congestion and sequestration (Scott & Ferguson, 1966; Powars, 1975). Subsequent episodes of recurrent vaso-occlusion and infarction lead to gradual auto-splenectomy, and in most patients the spleen is no longer palpable by the age of 10 years. In our cohort splenomegaly persisted until late childhood such that the spleen was still palpable in more than one-third of 10-year olds, an observation that is in accordance with previous studies conducted in sub-Saharan Africa (Esan, 1966; Adekile *et al*, 1988, 1993; Thuilliez & Vierin, 1997; Mouele *et al*, 1999). Some reports have attributed the persistence of splenomegaly in African children with SCD to the effects of malaria, although in this study we did not find an association with either the number of episodes of malaria or level of parasitaemia. A correlation has also been described between splenic size and immunoglobulin levels but there are conflicting data as to whether or not these antibodies are malaria-specific (Molineaux, 1988; Adekile *et al*, 1993). The majority of malaria occurs in children aged <6 years and, given the median age of our study population was 6·3 years, it is likely that only prospective follow up of a birth cohort will clarify any association between splenomegaly and malaria in children with SCD.

The relationship between SCD and malaria is complex, and remains the subject of some controversy. Malaria is widely believed to be a major cause of morbidity and mortality among children with SCD in Africa although the evidence for this is surprisingly limited, largely based on a small series in the 1970s (Molineaux *et al*, 1979). Malaria parasites do not grow well in red blood cells from individuals with SCD *in vitro*, at least under conditions of low oxygen tension (Friedman, 1978; Pasvol *et al*, 1978) and, on balance, the available data from epidemiological studies supports the conclusion that SCD may confer moderate protection against malaria (Okuonghae *et al*, 1992; Aluoch, 1997; Awotua-Efebo *et al*, 2004). This is supported further by the lack of evidence that widespread chloroquine resistance has resulted in an increase in mortality in SCD patients. At the time this study was conducted, Kilifi was meso-endemic for malaria with a typical prevalence of symptomless *Plasmodium falciparum* parasitaemia in the community of around 30% (O’Meara *et al*, 2008) – considerably higher than the prevalence observed in our cohort of patients with SCD (6%). The high compliance with anti-malarial chemoprophylaxis is probably one factor; however further studies are required to determine how much of this difference might be explained by protection by HbS and the particular properties of the red blood cells from children with SCD.

The mean haemoglobin concentration in children with SCD in our study (73 g/l) was similar to that reported in other parts of Africa (65–83 g/l) (Onyemelukwe, 1992; Ayatse, 1994; Diagne *et al*, 2000a; Rahimy *et al*, 2003). Higher values of 80–90 g/l have been reported from studies conducted in Jamaica and the USA (Serjeant *et al*, 1981; Thomas & Serjeant, 1997; Miller *et al*, 2000; Quinn & Ahmad, 2005), an observation that may be explained by the higher prevalence of iron deficiency found in Kilifi (Nyakeriga *et al*, 2005). Our observation that MCV was significantly raised in children with SCD is consistent with previous studies (Serjeant *et al*, 1981; Diop *et al*, 1999; Mouele *et al*, 1999), presumably due to the presence of a significant number of reticulocytes in the peripheral circulation. The mean MCHC (mean cell haemoglobin concentration) of 318 g/l is consistent with other reports from Africa but slightly lower than reported in Jamaica (Diop *et al*, 1999; Mouele *et al*, 1999). It is also significantly lower than that of normal children from the local population. The finding of a raised white blood cell count has been well-described in developed countries, and has been identified as a marker of severe SCD and, specifically, as a risk factor for early death, stroke, acute chest syndrome and nephropathy (Okpala, 2004). Whether the same will be true in this population is, as yet, unknown. The mean bilirubin concentration in patients in our cohort was raised at 46 μmol/l. In steady-state SCD, hyperbilirubinaemia is usually attributable to haemolysis rather than hepatic disease. Other tests of liver function are usually normal (Johnson *et al*, 1985; Omata *et al*, 1986), although derangements at older ages have been found in some studies (Green *et al*, 1953; Ferguson & Scott, 1959; Diggs, 1965). We are not aware of any previous reports of AST levels in steady-state African patients with SCD. One study found that lactate dehydrogenase levels were almost threefold higher than normal in adults and children living with SCD in the Congo (Mouele *et al*, 1999), although whether this was due to haemolysis or hepatic dysfunction is unclear. As expected, renal function tests were normal in all patients and the majority had a normal urine dipstick, although proteinuria and haematuria were detected in some older children. Ongoing follow up of this cohort will establish how these abnormalities evolve in later childhood.

In contrast to studies from developed countries, which have shown that from early life children with SCD are more stunted than normal children (Serjeant & Serjeant, 2001), we found no significant difference in height-for-age *Z*-scores between patients with SCD and local controls. In our study, however, the mean weight-for-age and weight-for-height *Z*-scores of children with SCD were both significantly lower than controls. This differs from a previous study in Nigeria which found no significant differences in height or weight of SCD and control children with the exception of a lower weight at 18 years of age (Oredugba & Savage, 2002). This highlights the importance of nutritional management in children with SCD living in an area where malnutrition is a major cause of morbidity and mortality (Maitland *et al*, 2006).

Patients with SCD are predisposed to hypoxaemia through a range of mechanisms including the lower affinity of HbS for oxygen, chronic anaemia, chest crises (Earley *et al*, 1998; Ohene-Frempong *et al*, 1998; Kirkham *et al*, 2001) and adenotonsillar hypertrophy (Ijaduola & Akinyanju, 1987; Wittig *et al*, 1988). In the developed world atopy may play an important role in nocturnal hypoxaemia, as allergic rhinitis and asthma are common in SCD (Boyd *et al*, 2004). There are, however, no data available for populations in Africa where chronic infection is common and atopy is rare. In this study we recorded hypoxaemia on only 6% of clinic visits, a figure which is low compared to previous studies, in which hypoxaemia has been reported in up to 54% of subjects (Samuels *et al*, 1992; Homi *et al*, 1997; Setty *et al*, 2003; Siddiqui & Ahmed, 2003). However, many of these studies were conducted during sleep and one included both adults and children. Performing a single measurement in the clinic is not the ideal way to measure hypoxaemia, and some subjects were hypoxaemic on multiple occasions. We therefore plan to carry out overnight oxygen saturation recording to obtain a more accurate estimate of the prevalence of nocturnal hypoxaemia in this population.

Few previous studies have documented hospital admission rates in African patients with SCD. A recent publication from Nigeria quoted annual admission rates as 1·21 and 0·04 per patient per year before and after implementation of a holistic care programme (Akinyanju *et al*, 2005), compared to the rate of 0·45 per patient per year described in our study. Historically, in many parts of Africa a marked deficit of adults with SCD has been noted relative to the numbers expected when considering the frequencies of the trait (Lambotte-Legrand & Lambotte-Legrand, 1955; Lehmann & Raper, 1956; Barclay *et al*, 1970; Ojwang *et al*, 1987), suggesting that, in the past, most affected individuals have died in childhood (Molineaux *et al*, 1979; Fleming, 1989). More recently, improved survival has been reported, and our mortality rate of 1% per year of follow up compares favourably to that reported in Benin (7%) (Rahimy *et al*, 2003) and Senegal (1·1%) (Diagne *et al*, 2000b).

Data from our cohort describe some of the similarities and differences between children living with SCD in Africa and the developed world. Describing the natural course of SCD in sub-Saharan Africa will increase awareness of the massive burden of disease and allow therapeutic interventions to be directed effectively and efficiently, in particular towards the prevention of malaria and severe anaemia and optimising nutritional status. It is imperative that these decisions are based on data from local populations rather than developed countries. Further follow up of this cohort, in addition to data from other parts of Africa, will help to provide the up to date information required, as well as baseline data for any intervention studies. In turn this will improve the outlook for the large number of children born with SCD in Africa every year.

## References

[b1] Adekile AD, Adeodu OO, Jeje AA, Odesanmi WO (1988). Persistent gross splenomegaly in Nigerian patients with sickle cell anaemia: relationship to malaria. Annals of Tropical Paediatrics.

[b2] Adekile A, McKie KM, Adeodu OO, Sulzer AJ, Liu JS, McKie VC, Kutlar F, Ramachandran M, Kaine W, Akenzua GI, Okolo AA, Asindi AA, Obinyan EA, Ogala WN, Ibrahim M, Huisman THJ (1993). Spleen in sickle cell anemia: comparative studies of Nigerian and U.S. Patients. American Journal of Hematology.

[b3] Akinyanju O, Otaigbe A, Ibidapo M (2005). Outcome of holistic care in Nigerian patients with sickle cell anaemia. Clinical and Laboratory Haematology.

[b4] Aluoch JR (1997). Higher resistance to *Plasmodium falciparum* infection in patients with homozygous sickle cell disease in western Kenya. Tropical Medicine and International Health.

[b5] Athale UH, Chintu C (1994). Clinical analysis of mortality in hospitalized Zambian children with sickle cell anaemia. East African Medical Journal.

[b6] Awotua-Efebo O, Alikor EA, Nkanginieme KE (2004). Malaria parasite density and splenic status by ultrasonography in stable sickle-cell anaemia (HbSS) children. Nigerian Journal of Medicine.

[b7] Ayatse E (1994). *Plasmodium falciparum* malaria: its effects on some haematological parameters in normal and sickle cell Nigerian children. Tropical Medicine and Parasitology.

[b8] Barclay GP, Jones HI, Splaine M (1970). A survey of the incidence of sickle cell trait and glucose-6-phosphate dehydrogenase deficiency in Zambia. Transactions of the Royal Society of Tropical Medicine and Hygiene.

[b9] Boyd JH, Moinuddin A, Strunk RC, DeBaun MR (2004). Asthma and acute chest in sickle-cell disease. Pediatric Pulmonology.

[b10] Corachan M, Oomen HA, Kigadye FC, Morris H (1979). Sicklers surviving childhood in Tanzania. Tropical and Geographical Medicine.

[b11] Diagne I, Ndiaye O, Moreira C, Signate-Sy H, Camara B, Diouf S, Diack-Mbaye A, Ba M, Sarr M, Sow D, Fall M (2000a). [Sickle cell disease in children in Dakar, Senegal]. Archives de Pédiatrie.

[b12] Diagne I, Soares G, Gueye A, Diagne-Gueye N, Fall L, N’Diaye O, Camara B, Diouf S, Fall M (2000b). [Infections in Senegalese children and adolescents with sickle cell anemia: epidemiological aspects]. Dakar Médical.

[b13] Diallo D, Tchernia G (2002). Sickle cell disease in Africa. Current Opinion in Hematology.

[b14] Diggs L (1965). Sickle cell crises. American Journal of Clinical Pathology.

[b15] Diop S, Thiam D, Cisse M, Toure-Fall A, Fall K, Diakhate L (1999). New results in clinical severity of homozygous sickle cell anemia, in Dakar, Senegal. Hematology and Cell Therapy.

[b16] Earley CJ, Kittner SJ, Feeser BR, Gardner J, Epstein A, Wozniak MA, Wityk R, Stern BJ, Price TR, Macko RF, Johnson C, Sloan MA, Buchholz D (1998). Stroke in children and sickle-cell disease: Baltimore-Washington Cooperative Young Stroke Study. Neurology.

[b17] Elamin AM (1980). Sickle cell anaemia in adult Zambian Africans. The Central African Journal of Medicine.

[b18] English M, Punt J, Mwangi I, McHugh K, Marsh K (1996). Clinical overlap between malaria and severe pneumonia in Africa children in hospital. Transactions of the Royal Society of Tropical Medicine and Hygiene.

[b19] Esan G (1966). The clinical spectrum of sickle cell disease in Nigerian adults. INSERM.

[b20] Ferguson AD, Scott RB (1959). Studies in sickle-cell anemia. XII. Further studies on hepatic function in sickle-cell anemia. AMA Journal of Diseases of Children.

[b21] Fleming AF (1989). The presentation, management and prevention of crisis in sickle cell disease in Africa. Blood Reviews.

[b22] Friedman MJ (1978). Erythrocytic mechanism of sickle cell resistance to malaria. Proceedings of the National Academy of Sciences of the United States of America.

[b23] Green TW, Conley CL, Berthrong M (1953). [The liver in sickle cell anemia.]. Bulletin of the Johns Hopkins Hospital.

[b24] Homi J, Levee L, Higgs D, Thomas P, Serjeant G (1997). Pulse oximetry in a cohort study of sickle cell disease. Clinical and Laboratory Haematology.

[b25] Ijaduola GT, Akinyanju OO (1987). Chronic tonsillitis, tonsillectomy and sickle cell crises. The Journal of Laryngology and Otology.

[b26] Johnson CS, Omata M, Tong MJ, Simmons JF, Weiner J, Tatter D (1985). Liver involvement in sickle cell disease. Medicine (Baltimore).

[b27] Kirkham FJ, Hewes DK, Prengler M, Wade A, Lane R, Evans JP (2001). Nocturnal hypoxaemia and central-nervous-system events in sickle-cell disease. Lancet.

[b28] Knox-Macaulay HH (1983). Sickle cell disease in Sierra Leone: a clinical and haematological analysis in older children and adults. Annals of Tropical Medicine and Parasitology.

[b29] Koko J, Dufillot D, M’Ba-Meyo J, Gahouma D, Kani F (1998). Mortality of children with sickle cell disease in a pediatric department in Central Africa. Archives de Pédiatrie.

[b30] Lambotte-Legrand J, Lambotte-Legrand C (1955). Le prognostic del’anemie drepanocytaire au Congo Belge (a propos de 300 case etde 150 deces). Annales de la Société Belge de Médecine Tropicale.

[b31] Lehmann H, Raper AB (1956). Maintainance of high sickling rate in an African community. British Medical Journal.

[b32] Maitland K, Berkley JA, Shebbe M, Peshu N, English M, Newton CR (2006). Children with severe malnutrition: can those at highest risk of death be identified with the WHO protocol?. PLoS Medicine.

[b33] Miller S, Sleeper L, Pegelow C, Enos LE, Wang WC, Weiner SJ, Wethers DL, Smith J, Kinney TR (2000). Prediction of adverse outcomes in children with sickle cell disease. New England Journal of Medicine.

[b34] Molineaux G (1988). The Garki Project – Research on the Epidemiology and Control of Malaria in the Sudan Savanna of West Africa.

[b35] Molineaux L, Fleming A, Cornille-Brogger R, Kagan I, Storey J (1979). Abnormal haemoglobins in the Sudan savanna of Nigeria. III. Malaria, immunoglobulins and antimalarial antibodies in sickle cell disease. Annals of Tropical Medicine and Parasitology.

[b36] Mouele R, Boukila V, Fourcade V, Feingold J, Galacteros F (1999). Sickle-cell disease in Brazzaville, Congo: genetical, hematological, biochemical and clinical aspects. Acta Haematologica.

[b37] Nyakeriga AM, Troye-Blomberg M, Chemtai AK, Marsh K, Williams TN (2004). Malaria and nutritional status in children living on the coast of Kenya. The American Journal of Clinical Nutrition.

[b38] Nyakeriga AM, Troye-Blomberg M, Mwacharo JK, Wambua S, Williams TN (2005). Nutritional iron status in children with alpha+ thalassemia and the sickle cell trait in a malaria endemic area on the coast of Kenya. Haematologica.

[b39] O’Meara WP, Mwangi TW, Williams TN, McKenzie FE, Snow RW, Marsh K (2008). Relationship between exposure, clinical malaria, and age in an area of changing transmission intensity. The American Journal of Tropical Medicine and Hygiene.

[b40] Ohene-Frempong K, Weiner SJ, Sleeper LA, Miller ST, Embury S, Moohr JW, Wethers DL, Pegelow CH, Gill FM (1998). Cerebrovascular accidents in sickle cell disease: rates and risk factors. Blood.

[b41] Ojwang PJ, Ogada T, Beris P, Hattori Y, Lanclos KD, Kutlar A, Kutlar F, Huisman TH (1987). Haplotypes and alpha globin gene analyses in sickle cell anaemia patients from Kenya. British Journal of Haematology.

[b42] Okpala I (2004). The intriguing contribution of white blood cells to sickle cell disease – a red cell disorder. Blood Reviews.

[b43] Okuonghae HO, Nwankwo MU, Offor E (1992). Malarial parasitaemia in febrile children with sickle cell anaemia. Journal of Tropical Pediatrics.

[b44] Omata M, Johnson CS, Tong M, Tatter D (1986). Pathological spectrum of liver diseases in sickle cell disease. Digestive Diseases and Sciences.

[b45] Onyemelukwe J (1992). Anti-thrombin III deficiency in Nigerian children with sickle cell disease. Possible role in the cerebral syndrome. Tropical and Geographical Medicine.

[b46] Oredugba F, Savage K (2002). Anthropometric finding in Nigerian children with sickle cell disease. Pediatric Dentistry.

[b47] Pasvol G, Weatherall DJ, Wilson RJ (1978). Cellular mechanism for the protective effect of haemoglobin S against *P. falciparum* malaria. Nature.

[b48] Powars D (1975). Natural history of sickle cell disease – the first 10 years. Seminars in Hematology.

[b49] Quinn C, Ahmad N (2005). Clinical correlates of steady-state oxyhaemoglobin desaturation in children who have sickle cell disease. British Journal of Haematology.

[b50] Rahimy M, Gangbo A, Ahouignan G, Adjou R, Deguenon C, Goussanou S, Alihonou E (2003). Effect of a comprehensive clinical care program on disease course in severely ill children with sickle cell anemia in a sub-Saharan African setting. Blood.

[b51] Samuels M, Stebbens V, Davies S, Picton-Jones E, Southall D (1992). Sleep related upper airway obstruction and hypoxaemia in sickle cell disease. Archives of Disease in Childhood.

[b52] Scott R, Ferguson A (1966). Studies in sickle cell anemia XXXVII: complications in infants and children in the United States. Clinical Pediatrics.

[b53] Serjeant G, Serjeant B (2001). Sickle Cell Disease.

[b54] Serjeant GR, Grandison Y, Lowrie Y, Mason K, Phillips J, Serjeant BE, Vaidya S (1981). The development of haematological changes in homozygous sickle cell disease: a cohort study from birth to 6 years. British Journal of Haematology.

[b55] Setty B, Stuart M, Dampier C, Brodecki D, Allen J (2003). Hypoxaemia in sickle cell disease: biomarker modulation and relevance to pathophysiology. Lancet.

[b56] Siddiqui A, Ahmed S (2003). Pulmonary manifestations of sickle cell disease. Postgraduate Medical Journal.

[b57] Snow RW, Schellenberg JR, Peshu N, Forster D, Newton CR, Winstanley PA, Mwangi I, Waruiru C, Warn PA, Newbold C, Marsh K (1993). Periodicity and space-time clustering of severe childhood malaria on the coast of Kenya. Transactions of the Royal Society of Tropical Medicine and Hygiene.

[b58] Thomas PW, Higgs DR, Serjeant GR (1997). Benign clinical course in homozygous sickle cell disease: a search for predictors. Journal of Clinical Epidemiology.

[b59] Thuilliez V, Vierin Y (1997). [The importance of sickle cell anemia in a pediatric environment in Gabon]. Sante Publique.

[b60] Urban BC, Cordery D, Shafi MJ, Bull PC, Newbold CI, Williams TN, Marsh K (2006). The frequency of BDCA3-positive dendritic cells is increased in the peripheral circulation of Kenyan children with severe malaria. Infection and Immunity.

[b61] WHO (2005). Integrated Management of Childhood Illness.

[b62] WHO (2006). Sickle-cell anemia. World Health Assembly Journal.

[b63] Wild B, Bain BJ, Lewis SM, Bain BJ, Bates I, Dacie JV (2006). Investigation of abnormal haemoglobins and thalassaemia. Dacie and Lewis Practical Haematology, 10th edition.

[b64] Williams TN, Mwangi TW, Wambua S, Peto TE, Weatherall DJ, Gupta S, Recker M, Penman BS, Uyoga S, Macharia A, Mwacharo JK, Snow RW, Marsh K (2005). Negative epistasis between the malaria-protective effects of alpha+-thalassemia and the sickle cell trait. Nature Genetics.

[b65] Wittig RM, Roth T, Keenum AJ, Sarnaik S (1988). Snoring, daytime sleepiness, and sickle cell anemia. American Journal of Diseases of Children.

